# Spatial-Temporal Changes and Associated Determinants of Global Heating Degree Days

**DOI:** 10.3390/ijerph18126186

**Published:** 2021-06-08

**Authors:** Yuanzheng Li, Jinyuan Li, Ao Xu, Zhizhi Feng, Chanjuan Hu, Guosong Zhao

**Affiliations:** 1 School of Resources and Environment, Henan University of Economics and Law, Zhengzhou 450046, China; yz_li@huel.edu.cn (Y.L.); ljy7872@163.com (J.L.); xuao2020@gmail.com (A.X.); fzz19990429@gmail.com (Z.F.); 2 Academician Laboratory for Urban and Rural Spatial Data Mining of Henan Province, Zhengzhou 450046, China; 3 Institute of Geographical Sciences, Henan Academy of Sciences, Zhengzhou 450052, China; huchanjuan1981@126.com; 4 School of Geography and Information Engineering, China University of Geosciences, Wuhan 430074, China

**Keywords:** climate change, energy consumption, thermal environment, Hurst exponents, influence factors, enhanced vegetation index, PM_2.5_, albedo, remote sensing, general regression neural network

## Abstract

The heating degree days (HDDs) could indicate the climate impact on energy consumption and thermal environment conditions effectively during the winter season. Nevertheless, studies on the spatial-temporal changes in global HDDs and their determinants are scarce. This study used multi-source data and several methods to explore the rules of the spatial distribution of global HDDs and their interannual changes over the past 49 years and some critical determinants. The results show that global HDDs generally became larger in regions with higher latitudes and altitudes. Most global change rates of HDDs were negative (*p* < 0.10) and decreased to a greater extent in areas with higher latitudes. Most global HDDs showed sustainability trends in the future. Both the HDDs and their change rates were significantly partially correlated with latitude, altitude, mean albedo, and EVI during winter, annual mean PM_2.5_ concentration, and nighttime light intensity (*p* = 0.000). The HDDs and their change rates could be simulated well by the machine learning method. Their RMSEs were 564.08 °C * days and 3.59 °C * days * year^−1^, respectively. Our findings could support the scientific response to climate warming, the construction of living environments, sustainable development, etc.

## 1. Introduction

The near-surface temperatures have increased significantly in most parts of the globe over the past 100 years, especially in recent decades [1,2]. During the 62 years from 1951 to 2012, the temperature has increased by 0.12 °C every ten years, which was 1.88 times the increase observed since 1880 [1]. Climate change can cause consequences that are not only related to the energy consumption for heating and cooling but also the indoor and outdoor thermal comfort conditions, and can further affect human health, especially that of sick people, the elderly and children, etc. [3,4]. Buildings are among the sectors with the highest energy consumption, accounting for approximately 40% of the total global energy consumption and generating more than 30% of human-made carbon dioxide emissions [5]. A considerable part of this energy consumption is used in space heating and cooling. Heating, ventilation, and cooling account for 35% of primary energy use in America, while a similar level will be reached within five years in China [6]. High energy consumption could result in huge emissions of greenhouse gases and harmful gases, the depletion of abiotic resources, acid precipitation and acidification, the depletion of stratospheric ozone, ecotoxicity and radiological exposure, and the health problems they can cause, etc. [7,8]. In the future, climate change will have more significant impacts on energy consumption and thermal comfort conditions with the increasing global population [7], rapid progression of global urbanization, the increase in ownership and utilization rates of air conditioning [6,9], the improvements in people’s economic conditions [10] and living standards [7], the popularity of glass facade buildings [11], and so on.

It is of great theoretical and practical value to analyze climate change’s influence on energy consumption and thermal comfort conditions. Building heating and cooling energy consumption is generally assumed to be proportional to the difference between indoor and outdoor temperatures. Based on this assumption, the degree days indices, which are closely related to temperature, have become a widely recognized and adopted simple, efficient, and reliable indicator to represent the impacts of climate change on the energy consumption of building heating and cooling [8,12,13,14]. Moreover, some studies used the degree days indices to indicate the thermal comfort conditions [12,13,14,15]. These indices can be mainly divided into three categories: heating degree days (HDDs), cooling degree days (CDDs), and their sum (HDDs + CDDs) [8,12,13,14]. Previous studies have analyzed the spatial-temporal changes in HDDs or CDDs [8,13,14], HDDs + CDDs [15] in the past [8,12,14] or the future [15,16,17] at the city [18,19], region [13,20] or global scale [8,12,14]. Moreover, some studies have also explored the influence factors. The current study areas are limited to a tiny number of places, including Madison, WI, USA [21], Florence, Italy [22], Andalusia Autonomous Region, Spain [23], Bangladesh [13], Xinjiang Uygur Autonomous Region, China [24], etc. Factors that have been considered include latitude, longitude, and altitude [13,24], distance to large water bodies [21,23], the abundance of impervious surfaces [21,22], large-scale atmospheric circulation indices [13], etc. However, to the best of our knowledge, studies on the spatial-temporal changes and the associated determinants of global HDDs are scarce, which is very important for estimating energy consumption, the indoor and outdoor thermal environment, and human’s public health in the winter season. Therefore, our study aimed to use multi-source data and several methods to explore the rules of spatial distribution and interannual changes and some important influencing factors of global HDDs.

## 2. Materials and Methods

### 2.1. Materials

The global HDD data at a 0.25° × 0.25° gridded resolution, spanning 90° N–60° S, covering 49 years over the period 1970–2018, were provided by the official website of PANGAEA (a data publisher for earth & environmental science) (https://doi.pangaea.de/10.1594/PANGAEA.903123) (accessed on 2 June 2021) [12]. They were derived using meteorological variables from the Global Land Data Assimilation System (GLDAS) [25]. GLDAS is a new generation global high-resolution reanalysis data product developed jointly by the National Aeronautics and Space Administration, Goddard Space Flight Center, and National Centers for Environmental Prediction [26]. GLDAS provides a consistent quality-controlled long global gridded time series of several key meteorological variables at fine-scale spatial-temporal (0.25° gridded, 3-hourly) resolution [26]. Equation (1) shows the calculation method of HDDs.

(1) $$HDDs=\sum_{i=1}^{n}{\left(T_{b}-T_{d}\right)}^{+}$$

where ‘+’ signifies that only positive values accumulate over *n* days in the chosen period, and $T_{d}$ and $T_{b}$ represent the daily mean outdoor air and base (threshold) temperatures, respectively. The $T_{b}$ was defined as 18 °C in this study, referring to previous studies [12] and increasing the standard of living thermal comfort.

The global monthly vegetation indices data (MYD13C2.006) and global daily albedo data (MCD43C3.006) at 0.05 degree (5.6 km at the equator) and the global land cover data (MCD12Q1.006) with the spatial resolution of 1 km in 2016 were downloaded from the official website of moderate resolution imaging spectroradiometer (MODIS) (https://search.earthdata.nasa.gov/search) (accessed on 2 June 2021). In generating this monthly product, the algorithm ingests all the MYD13A2 products (with the spatial resolution of 1 km and the temporal resolution of 16 days) that overlap the month and employs a weighted temporal average. The enhanced vegetation index (EVI) was adopted in this study because EVI has improved sensitivity over high biomass regions than the normalized difference vegetation index. The global annual visible infrared imager radiometer sensor (VIIRS) nighttime light (NTL) data at the spatial resolution of 15 arc seconds (about 0.5 km at the equator) in 2016 were obtained from the website of the Colorado School of Mines (https://eogdata.mines.edu/download_dnb_composites.html) (accessed on 2 June 2021). The global urban extent data in 2016 with the spatial resolution of 250 m were provided by the official website of the Institute of Remote Sensing Information Processing, Wuhan University (http://irsip.whu.edu.cn/resources/MGUP.rar) (accessed on 2 June 2021) [27]. These urban regions were derived from the MODIS land cover type produce (MCD12Q1) based on a locally adaptive and fully automated global mapping approach. Accuracy assessment indicates that MGUP has an F-score of 0.88, achieving better results than the contemporary global products. The annual PM_2.5_ data were downloaded from the official website of Atmospheric Composition Analysis Group, Washington University (http://fizz.phys.dal.ca/~atmos/martin/?page_id=273) (accessed on 2 June 2021). They were derived using advances in satellite observations, chemical transport modeling, and ground-based monitoring [28]. The resultant annual mean geophysical PM_2.5_ estimates were highly consistent with globally distributed ground monitors (R_2_ = 0.81, slope = 0.90) [28]. Other data included the global digital elevation model data with the spatial resolution of 30 s (about 0.5 km) in ENVI 5.3, global continents border in ArcMap 10.2, etc.

### 2.2. Methods

#### 2.2.1. Spatial-Temporal Changes in Global Heating Degree Days

First, the global average HDDs during the last five years (2014–2018) were computed. Second, the change rates of global HDDs from 1970 to 2018 in each grid and their corresponding significance levels were calculated using the widely applied Mann–Kendall test method, which did not require the sample data to fit a certain distribution type and was not disturbed by a few abnormal values [12,24,29,30,31]. Third, the global spatial autocorrelation analysis of HDDs and their interannual change rates were done by using Moran’s I statistic method [32]. When Moran’s I was larger or smaller than 0, the HDDs and their change rates had positive or negative spatial correlation, respectively; when Moran’s I was equal to 0, both the HDDs and their interannual change rates were distributed randomly and did not have an obvious spatial correlation. Moreover, the degrees of clustering of either high or low values of HDDs and their interannual change rates were measured using the Getis-Ord General G statistic. When the derived z-scores were larger or smaller than 0, the HDDs or their interannual change rates had high or low values aggregation areas. In addition, local Moran’s I analysis (LISA) was performed for global HDDs at the grid-scale, which can identify the spatial heterogeneity and the dependence of each spatial object attribute in local space [32]. Four types of clusters can be obtained through LISA analysis, including high-high (HH) clusters, low-low (LL) clusters, high-low (HL) clusters, and low-high (LH) clusters. The first and latter two areas corresponded to the spatially dependent and heterogeneous areas, respectively. In addition, the hot spot analysis was done for the interannual rates of HDDs. From this, the clusters of both high or low significant values can be derived. Finally, the Hurst exponent (*H*) approach through rescaled range (R/S) analysis was adopted to explore the variation trends of global HDDs in the future. It is an effective method to describe the self-similarity and long-term dependence of climate [33], hydrology [34], environmental factors [35], etc. The computed Hurst exponents derived by the R/S analysis method were more reliable and robust than other approaches [33]. If *H* equals 0.5, the time series of HDDs is uncorrelated and could be described as a random walk; if *H* is large than 0.5 and smaller than 1, the time series is sustainable and characterized by long-term correlation; if *H* is larger than 0.5 and smaller than 1, the time series is anti-sustainable and characterized by the opposite variation trends in the future compared with the past [33,34,35]. Furthermore, the sustainability types of variation trends of time series could be divided into weak (0.5 < *H* < 0.75), medium (0.75 < *H* < 0.85) and strong sustainability (0.85 < *H* < 1); and the anti-sustainability types of variation trends of time series could be categorized as weak (0.35 < *H* < 0.5), medium (0.25 < *H* < 0.35) and strong anti-sustainability (0 < *H* < 0.25) [36].

#### 2.2.2. Associated Determinants of Global Heating Degree Days and Their Interannual Change Rates

Seven influence factors were analyzed for HDDs and their interannual change rates, including the latitude, altitude, distance to large waterbodies, mean enhanced vegetation index in winter, mean albedo in winter, annual PM_2.5_ concentration, and nighttime light intensity. Latitude refers to the absolute value of latitude in this study. The distance to large waterbodies was defined as the Euclidean distance to the global coastline and the lake shoreline of the Caspian Sea (the largest lake in the world). To overcome the spillover effects of waterbodies, these VIIRS NTL intensities were reset as 0 in the waterbodies. Moreover, we set the abnormal values as 0, including the negative and extremely large values, which referred to the NTL intensities were larger than their largest values in the urban areas. The two largest urban agglomerations were chosen to determine the largest NTL intensity in each continent except for Antarctica.

The partial correlation analysis method can analyze the relationship between two variables while controlling for the linear influences of other variables. Therefore, it can reflect the internal relationship between the two variables more truly than correlation analysis. The partial correlation coefficients and their significance levels were computed between HDDs in 2016 or their interannual change rates from 1970 to 2018 and the seven associated determinants mentioned above.

Moreover, we built the stepwise multiple regression equations and computed both the determination coefficients and their corresponding significant levels. In addition, both HDDs and their interannual change rates were simulated by the general regression neural network (GRNN) algorithm, which exhibits a strong nonlinear mapping ability, high fault tolerance, and robustness [37]. Moreover, it can obtain good fitting and prediction results when the number of samples is small or instability exists in the data [37]. This method has been universally applied to various prediction and forecasting tasks in civil engineering and environmental science disciplines [31,38,39]. The proportion was set as 4:1 between training and testing sample sets. The root-mean-square error (RMSE), correlation coefficient (R), and the corresponding significance level were calculated to indicate the simulation accuracy. The parameter optimization and simulation processes were repeated five times to avoid uncertainties during the process and to obtain stable results. It should be noted that the operations mentioned above in this paragraph have been performed only when the HDDs existed, which means the HDDs were larger than 0 during the past 49 years.

## 3. Results and Discussion

### 3.1. Spatial-Temporal Changes in Global Heating Degree Days

#### 3.1.1. Spatial Variations of Global Heating Degree Days

**Figure 1:**
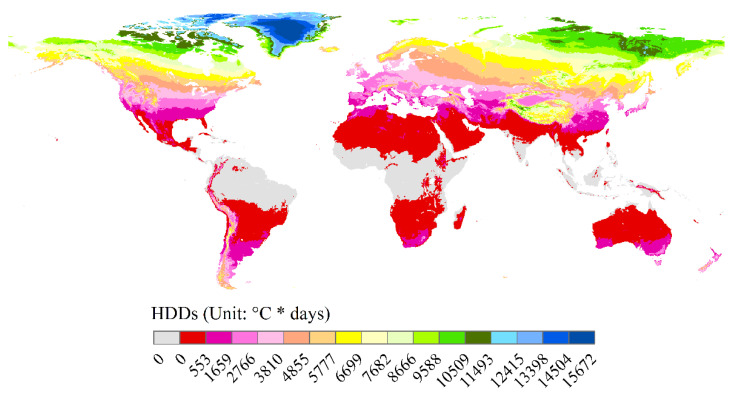
Distribution of global heating degree days between 2014 and 2018.

The global five-year average HDDs showed extremely obvious spatial variation laws, which generally became larger in places with higher latitudes and altitudes (Figure 1). HDDs did not exist in most regions which the equator crossed, including northern and central South America, central Africa, and South and Southeast Asia. The HDDs were lower than 553 °C * days in the regions surrounding the regions mentioned above—in the vast majority of Central America, central South America, northern and southern Africa, the Arabian peninsula, central and northern India, northern Southeast Asia, and central and northern Australia, etc. The HDDs in Qinghai-Tibet Plateau were obviously lower than its nearby regions, most ranging from 2766 to 11,493 °C * days. The largest HDDs occurred in central Greenland, at larger than 14,504 or even 15,672 °C * days.

**Figure 2:**
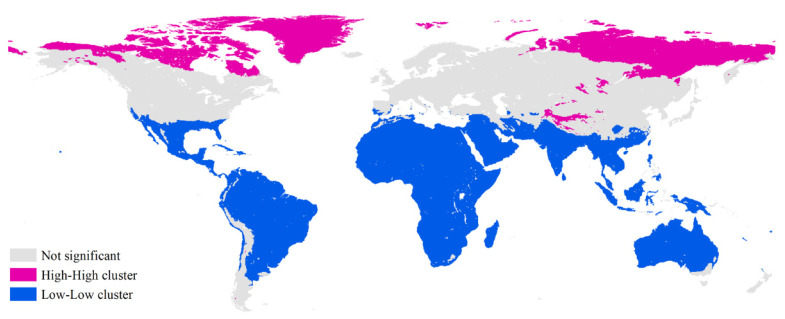
Spatial pattern of global heating degree days based on Anselin Local Moran Index.

The global Moran’s index for global five-year average HDDs was 0.988 at the 0.000 significant level. This indicated that high spatial positive correlations existed for global HDDs, and their values became more similar as the measured distance decreased. Moreover, both HH and LL clusters existed (Figure 2). The HH clusters mainly occurred in Greenland, northern Canada, northeastern Siberia, and some regions in Qinghai-Tibet Plateau and Sayan Mountains (a large upland area lying along the frontiers of east-central Russia and Mongolia). The LL clusters were mainly distributed in the southern United States, Central America, to vast majority of central South America, Africa, the Arabian peninsula, most of Iran, southwestern Afghanistan, south and southeast Asia, some of southern China, and the overwhelming majority of Australia.

#### 3.1.2. Interannual Spatial-Temporal Changes in Global Heating Degree Days

**Figure 3:**
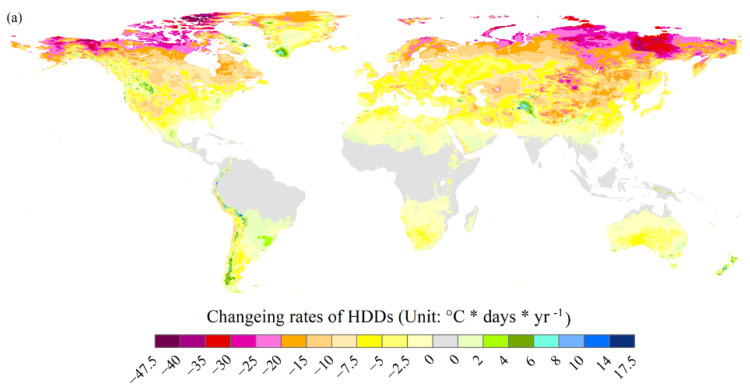

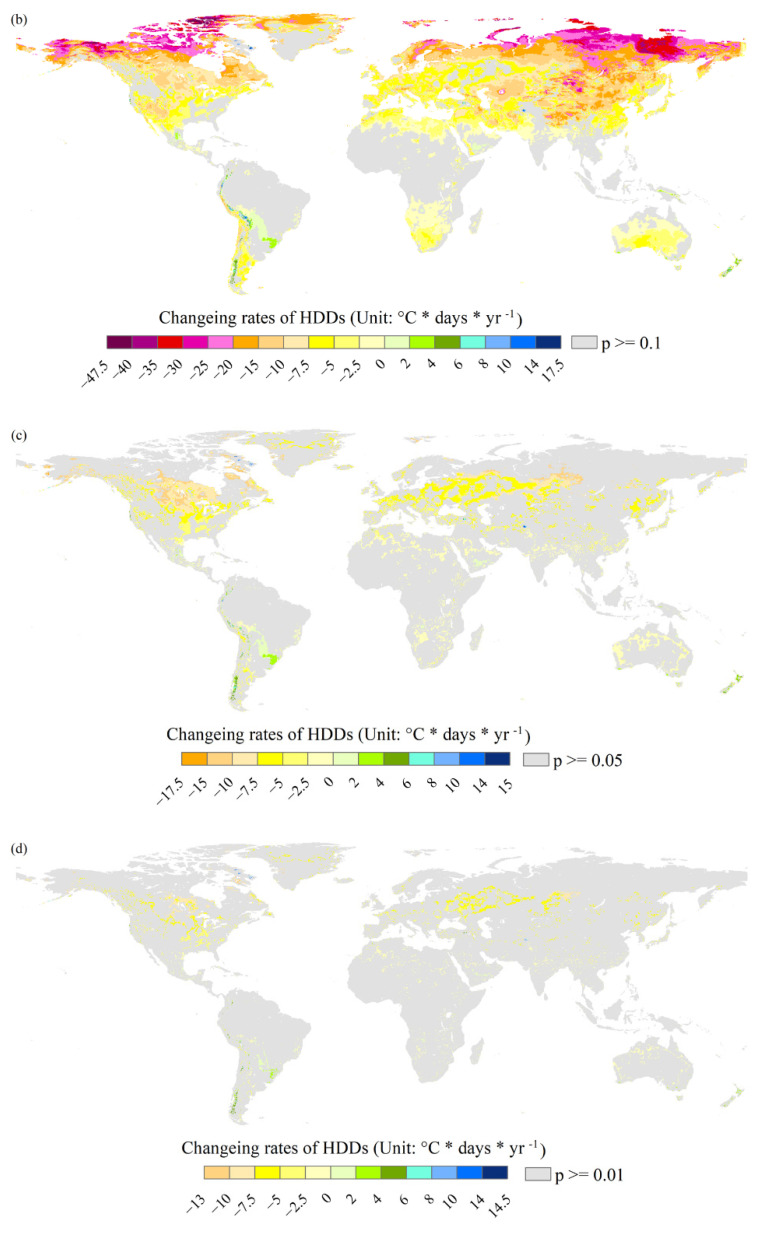
Change rates of global heating degree days (HDDs) from 1970 to 2018. (**a**) The significant levels of change rates of HDDs are not considered. (**b**–**d**) Only change rates at the 0.1, 0.05, and 0.01 levels are considered, respectively.

Most global change rates of HDDs were negative during the past 49 years (throughout 1970–2018) due to the unequivocal global warming in most regions of the world (Figure 3a) [1]. The rates of HDDs generally decreased to a greater extent in areas with higher latitudes due to their larger increase rates of near-surface air temperatures [1]. The decreasing rates ranged from −7.5 to 0 °C * days * year^−1^, and were mainly distributed in the southeastern United States, a few regions in the western part of Canada and the United States, most of Greenland, most of the southern tip of South America, most of Europe except for northern Europe, and some of the surrounding West Siberian Plain areas, northern and southern Africa, most of West Asia, northern South Asia, southeastern China, northeastern China, Outer Manchuria, the Korean peninsula, and Japan. The declining rates ranging from −20 to −7.5 °C * days * year^−1^, and mainly included most of northeastern Canada, some of the western United States and northern Greenland, northern Europe and its nearby Russian regions, some regions located to the east of Caspian Sea, northern China (including Tibet, but excluding northeastern China), Mongolia, and some areas in some central and eastern Siberian regions. The largest change rates were chiefly located in Alaska, northern Canada, quite a few northern Russian and some central Siberian regions, and some western Mongolian regions. It should note that HDDs did not decrease and instead increased in some areas. The change rates were mainly from 0 to 4 °C * days * year^−1^ in these regions, mainly including most of southeastern South America, certain areas in Mexico, certain regions in northern Africa, the Arabian peninsula, northern India, southwestern China and Hainan island, northeastern regions of Southeast Asia, southeastern Australia and some regions in New Zealand, and a few regions in the northwestern, eastern and southeastern United States, Greenland, southwestern Norway, western Ukraine, southern Africa, eastern Turkey, and northern and western Australia. Moreover, the change rates were from 6 to 8 °C * days * year^−1^ in some places, including some regions in Greenland, Central Asia, and north India adjacent to China, a few regions in the northwestern United Stated, the southern Andes, and New Zealand. In addition, the variation rates can be larger than 8 or can even be 17.41 °C * days * year^−1^ in a few regions in northeastern marginal areas of Canada, the northern Andes, and eastern marginal areas of Afghanistan.

The change rates mentioned above in most regions passed the significant level of 0.1, especially in the middle and higher latitudes (Figure 3b). The areas with the variation rates at the significance level of 0.05 mainly included most regions in central and eastern Canada and their adjacent regions in the United States, many European regions and a certain number of areas in neighboring Russia and Kazakhstan, and some regions in South America (Figure 3c). The areas with change rates at a significance level of 0.01 continued to decrease. These areas were mainly distributed in regions in the center of Canada and the United States, eastern Europe and its neighboring regions in Russia and Kazakhstan, and South America (Figure 3d).

**Figure 4:**
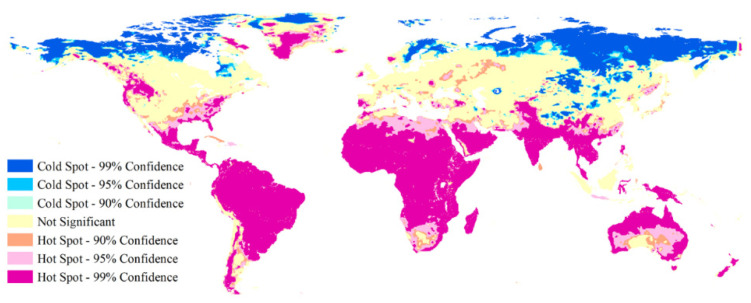
Spatial pattern of change rates of global heating degree days from 1970 to 2018 based on hotspot analysis.

The global Moran’s index for the change rates of global HDDs during the last 49 years was 0.946 at the 0.000 significant level. This indicates that high spatial positive correlations existed for the variation rates of global HDDs. Moreover, both cold and hot spots at the 0.10, 0.05, and 0.01 significant levels existed (Figure 4). The cold spots with high confidence referred to regions with change rates of HDDs that were obviously smaller than other regions due to their evidently larger increasing rates of air temperature. These regions at the significance of 0.05 mainly occurred in northern marginal areas in North America and northern Europe, and most of central and eastern Siberia, and some regions in western Mongolia and its surrounding Russian regions, some areas in the regions where northeastern China, Russia and Mongolia were adjacent, and some regions in the Qinghai-Tibet Plateau, the northwestern Loess Plateau, and the western section of the South Tianshan Mountains. The hot spots with high confidence referred to regions with change rates of HDDs that were obviously larger than other regions due to their obvious smaller air temperature increasing rates. These regions at the significance of 0.05 mainly included the vast majority of Central and South America, the eastern, southeastern and northwestern United States, western Canada, central and southern Greenland, the overwhelming majority of Africa, western Asia, most of South and Southeast Asia, and some their surrounding regions in central Asia and southern China, as well as the vast majority of Oceania.

**Figure 5:**
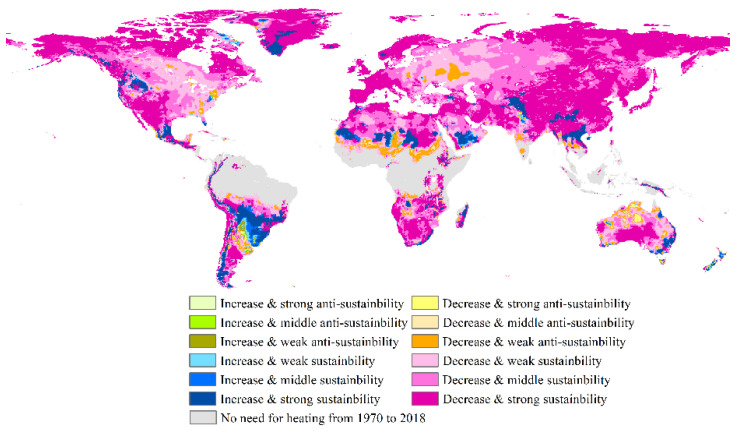
Types of trends in the past and future of global cooling degree days based on the Mann–Kendall test and Hurst exponent methods.

Most global HDDs decreased in the past and showed sustainability trends in the future (Figure 5). The strong sustainability type with decreased trends was widely distributed in northern North America, the southwestern United States and some of its adjacent northwestern Mexico regions, North and East Europe, northern and southern Africa, most of the northern Arabian peninsula, Iran and Afghanistan, certain areas in Pakistan and its surrounding Indian regions, the vast majority of the regions in northeastern India, East and Northeast Asia, and many regions in central and southern Australia. The medium sustainability type with decreased trends was concentratedly distributed in the surrounding regions of the above-mentioned type, especially in the West Siberian Plain and central Asia. The weak sustainability type with decreased trends was mainly distributed in eastern North America, East Europe, and Australia. Nevertheless, there were a few regions with decreased rates but anti-sustainability trends (Figure 5). Those regions with strong anti-sustainability were mainly located in some Australian regions. Those regions with weak anti-sustainability were distributed primarily in the southern marginal areas of North Africa, some of southwestern Russia, some of India, and southeastern Asian regions, as well as a few regions in Australia. The HDDs increased in some areas over the past 49 years and showed strong sustainability, mainly including a few regions in southeastern South America, the Andes, central Mexico, the northwestern United States, the western marginal areas of Canada, southern and central Greenland, some regions in northern Africa and the Arabian peninsula, Tajikistan, northeastern Pakistan, northern India and its nearby regions, some Southeast Asian regions and some their surrounding southern Chinese regions, some regions in western marginal Australia and New Zealand. Overall, the future change type was sustainability in the majority of global regions (Figure 5), meaning the change direction will continue in these regions in the future.

### 3.2. Associated Determinants of Global Heating Degree Days

#### 3.2.1. Associated Determinants of Spatial Variation of Global Heating Degree Days

**Figure 6:**
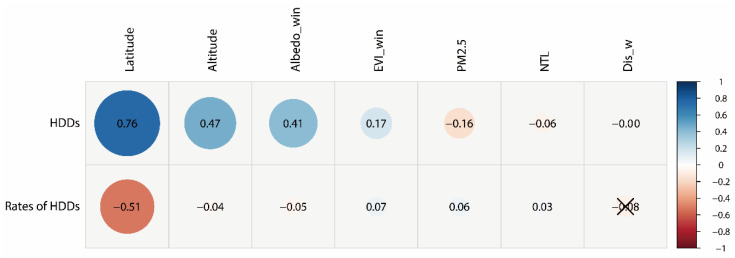
Partial correlation coefficients between the influence factors and heating degree days (HDDs) and their interannual change rates. The Albedo_win, Dis_w, EVI_win, PM_2.5_, and NTL corresponded to the mean albedo during winter, distance to large waterbodies (seas or oceans), mean EVI during winter, annual NTL intensity, and annual mean PM_2.5_ concentration, respectively. The “×” indicates that no significant partial correlation existed (*p* > 0.05).

The HDDs were significantly positively partially correlated with latitude, altitude, mean albedo, and EVI during winter (*p* = 0.000) (Figure 6). The absolute value of latitude has obvious effects on HDDs (r = 0.76) due to the lower incoming solar radiation at higher latitudes. The HDDs could be larger with the increase in altitude (r = 0.47) because of the lower absorption of longwave radiation, the poorer heat-retaining capacity of the thinner air, etc. Previous studies have found significant correlations between HDDs and latitude, HDDs and altitude [13,24]. The partial correlation coefficient was 0.41 between HDDs and the mean albedo during winter. This was because materials with lower albedos could absorb more energy during the daytime. The stored heat can be emitted upward into the atmosphere during the nighttime, contributing to higher ambient temperatures, as stated in previous studies [40,41,42]. The HDDs were positively partially correlated with the mean EVI during winter (r = 0.17, *p* = 0.000). This is mainly because higher vegetation activity could lead to the decrease in air temperatures and the increase in HDDs during the daytime due to the impact of the transpiration of plants. However, it should be noted that more and denser vegetation (especially in forests) may increase air temperature and lower HDDs during the nighttime. On the one hand, the penetrated solar energy could be stored within and beneath canopies because of the water content [43]. On the other hand, more and denser vegetation could decrease the sky view factor, lowering the amount of radiation emitted into the open sky [43]. Moreover, a previous study found that temperate forests show moderate warming in winter, and that boreal forests have strong warming in winter and net warming annually due to the lower albedo than nearby open land (grassland and cropland) [44]. This means that higher and denser vegetation could decrease the air temperature and further increase HDDs from the all-day aspects at the global scale.

The annual mean concentration of PM_2.5_ was significantly negatively partially correlated with HDDs (r = −0.16, *p* = 0.000). This result is consistent with previous related studies, which found that the annual surface urban heat islands during the nighttime could be obviously enhanced by haze pollution in China [45], and that aerosols could increase the urban heat island during the winter daytime in China [46]. The NTL intensities are usually used to indicate the intensities of human activities and are closely related to the abundance of impervious surfaces. In theory, higher NTL intensities could lead to an increase in temperature and a decrease in HDDs. The effect was minor in this study (r = −0.06, *p* = 0.000). Nevertheless, the abundance of impervious surface could explain 67% of the spatial variation of HDDs in Madison, Wisconsin, United States [21]. This difference may be due to different indicators, especially different research scales. Another important potential reason for this was that HDD datasets were derived from reanalysis data, whose validity was controversial in analyzing the effects of urbanization on temperature (the most important determinant of HDDs) [47,48]. In theory, the distance to large waterbodies has a positive impact on HDDs. This is because it could be warmer in those regions closer to large waterbodies. Nevertheless, this effect was neglected in this study (r = −0.00). This may be related to the complex atmospheric circulations and ocean currents, the neglect of the effects of other large waterbodies, topographic obstructions, etc.

The multiple regression equation between HDDs and their seven influence factors is shown in Equation (2). The explain rate was 85.30%, which indicated the HDDs could be simulated by these determinants at any place where the temperature data were not accessible or available. The predicted HDDs by GRNN algorithm have high accuracy (Table 1). The RMSEs for the training and testing samples were 551.59 and 564.08 °C * days, respectively. Moreover, the simulated HDDs were significantly highly correlated with the actual HDDs (Table 1). The correlation coefficients were 0.987 and 0.986 for the training and testing samples at the significance level of 0.000, respectively.

(2) $$\begin{array}{ll} HDD= & 125.67*Lat+0.94*Alt+5429.53*Alb\_win+2556.15*EVI\_win-39.73*\mathrm{NTL}-13.24*PM_{2.5}\\ & -3752.86(R^{2}=0.8530,\;p=0.000) \end{array}$$

where *Lat*, *Alt*, *Alb_win*, *EVI_win*, *PM*_2.5_, and *NTL* correspond to the absolute values of latitude, altitude (with the unit of meters), mean albedo and *EVI* during winter, annual mean *PM*_2.5_ concentration and *NTL* intensity, respectively.

#### 3.2.2. Associated Determinants of the Interannual Change Rates of Global Heating Degree Days

The change rates of HDDs were significantly partially correlated with the seven influence factors mentioned above (*p* = 0.000), except for the distance to large waterbodies (*p* = 0.388) (Figure 6). The regions with higher latitude experienced higher decrease rates (r = −0.51). The main reason for this was the obviously larger increase in temperature in these regions, especially in the Northern Hemisphere [2]. Moreover, the increased temperatures could easily contribute to the accumulation of HDDs due to the lower temperatures here, which were usually lower than the base temperature for heating. Therefore, the significant cold spots were mainly located in the northernmost regions. In contrast, the hot spots were densely distributed in the areas with lower latitude and their surrounding areas, especially in the Southern Hemisphere. These spatial patterns of the change rates of temperature and HDDs were probably because the widely distributed ice and snow in higher-latitude regions were more sensitive to climate change. The melted ice and snow could decrease the albedo, increase the moisture content in the air and the clouds in the atmosphere, which could reduce the reflection of short-wave radiation, increase the absorption of long-wave radiation in the air, and enlarge the sunshine duration, etc. In addition, the convection effects were weak in high-latitude areas, and the near-surface heat was difficult to diffuse into the upper atmosphere. All the factors mentioned above could accelerate the temperature rise. The effects of altitude on the variation rates of HDDs were negative but small (r = −0.04) at the global scale. On the one hand, the contributions of increased temperatures on HDDs were more obvious due to the lower temperatures in the regions with higher latitude, which were usually lower than the base temperature for heating. On the other hand, the relationships between the attitude and the variation rates of temperature [49], or HDDs, were complex in different locations and contexts. For instance, the rates of HDDs were larger and smaller than its surrounding regions with lower altitudes in the Qinghai-Tibet Plateau and some Andean regions, respectively. Areas with higher albedo were more sensitive to climate warming and gained higher and smaller change rates of air temperature and HDDs (r = −0.05), respectively.

Regions with higher and denser levels of vegetation were more insensitive to climate change, mainly due to the adjustment abilities of vegetation on temperature [50,51], resulting in lower variation rates of temperature and HDDs in these regions. However, the partial correlation coefficient was minor (r = 0.07) between the change rates of HDDs and mean EVI during winter. This result was mainly due to the lower coverage and activities of vegetation during the cold period. Moreover, positive partial correlations existed between the variation rates of HDDs and PM_2.5_ concentration (r = 0.06). This result may be because of the larger greenhouse effect that existed in the polluted air, resulting in lower change rates of temperature and HDDs. In addition, the NTL intensity had minor significant positive impacts on the variation rates of HDDs (r = 0.03). The larger NTL intensity meant higher urbanization levels. A previous study found that nighttime surface urban heat island intensities (SUHIIs) became larger in some cities (*p* < 0.05) from 2003 to 2013 in China during the winter, while the daytime SUHIIs became larger and smaller in some cities (*p* < 0.05) in southern and northern China, respectively [52]. This finding could indicate complex relationships between the NTL intensity and the change rates of HDDs to a certain degree. Theoretically, the regions closer to large waterbodies were liable to enjoy lower variation rates of HDDs due to the larger regulating effects of the seas or oceans on temperature. However, no significant correlations existed in this study. This may be due to the complexity of atmospheric circulations and ocean currents, the neglect of other large waterbodies, topographic obstructions, etc. The multiple regression equation between the interannual change rates of HDDs during the past 49 years and their seven influence factors is shown in Equation (3). The explanatory rate was 58.81%, which indicates that the interannual variations rates of HDDs could be partially simulated by these determinants in any region where the temperature data were not accessible or available. The predicted change rates of HDDs by GRNN algorithm have high accuracy (Table 1). The RMSEs for the training and testing samples were 3.56 and 3.59 °C * days * year^−1^, respectively. Moreover, the simulated change rates of HDDs were significantly highly correlated with the actual rates (Table 1). The correlation coefficients were 0.881 and 0.879 for the training and testing samples at the significance level of 0.000, respectively.

(3) $$\begin{array}{ll} V_{HDD}= & -0.24*Lat-0.0004*Alt-2.99*Alb\_win+3.92*EVI\_win+0.09*\mathrm{NTL}+0.01*PM_{2.5}\\ & +2.75(R^{2}=0.5881,\;p=0.000) \end{array}$$

where *V_HDD_* refers to the interannual change rates of HDDs, and the meanings of *Lat*, *Alt*, *Alb_win*, *EVI_win*, *NTL*, *PM*_2.5_ are the same as in Equation (2).

#### 3.2.3. Limitations and Future Work

The datasets of HDDs were derived from the reanalysis data, which were based on the upper air observations and did not directly include the effects of the local surface on observations [53,54,55]. Moreover, some drawbacks existed regarding the reanalysis data, including the neglect of clouds and surface moisture, changes in atmospheric composition effects [54], non-homogenization and time-varying of the input data [56], and bias associated with the income and expense balance of surface heat [54]. Therefore, although this study was able to obtain certain findings on the spatial-temporal changes and associated determinants of global HDDs, some uncertainty still existed. Exploring these laws using other data, especially the near-surface observed records, should be encouraged in the future.

Due to the coarse resolution of HDDs, the large research areas (the global regions), and the difficulty of data availability, etc., some important influence factors have not been considered in this study, such as the greenhouse gas concentration, atmospheric circulation indices, landscape composition and pattern, remotely sensed indices of buildings, water and bare soil, the sky view factor, heat release, population density, etc. [13,57]. Moreover, only the effects of seas, oceans, and the Caspian Sea on HDDs and their interannual change rates were considered in this study, rather than all large waterbodies. This was mainly because it was too difficult to determine the influence distances of waterbodies with different sizes [31] and to quantify the combined effects of several waterbodies. In addition, the annual PM_2.5_ concentration was used rather than the mean value in winter due to the lack of data availability. In addition, it should be noted that only the data in a single year rather than several years were adopted to explore the influences of the distribution of HDDs. However, this may not be a limitation but an advantage, since using the mixed data over many years is likely to reduce the correlation degrees between HDDs and their factors. Moreover, scholars should be encouraged to analyze the change rates of HDDs with the variation rates of some determinants rather than their mean values during the specified period. This has not been analyzed in this study mainly due to the data shortage of many determinants covering such a long period, 1970–2018. In addition, the driving forces for HDDs should be analyzed under different contexts at multiple scales during different periods (such as in the different climate zones during the daytime and nighttime at the local, regional and global scales, etc.), due to their spatial-temporal heterogeneity under different conditions at different scales. In addition, although the driving forces of spatial-temporal changes in HDDs and their interannual change rates were analyzed by several methods, including partial correlation analysis, multiple regression, and machine learning, more accurate and detailed mechanisms should be explored by more methods, such as experimental observation, numerical simulation, etc.

## 4. Conclusions

This study aimed to use the climate data, remotely sensed data, PM_2.5_ concentration data, etc., and adopted the spatial analysis, mathematical statistics, and machine learning method, etc., to explore the rules of spatial distribution and their interannual changes in global HDDs, and some important influencing factors. The main findings were as follows:

(1) The global HDDs showed extremely obvious spatial distribution laws, which generally became larger in places with higher latitudes and altitudes. The largest HDD was 15,672 °C * days, which occurred in central Greenland. High spatial positive correlations existed for global HDDs, and both HH and LL clusters existed.
(2) Most global change rates of HDDs were negative during the past 49 years (over the period 1970–2018), and they generally decreased to a greater extent in areas with higher latitudes. The negative rates mainly occurred in southeastern South America, the Andes, Mexico, the northwestern United States, southern Greenland, southern North Africa, the southern Arabian peninsula, eastern Turkey, northern South Asia, and its northern surrounding regions, northern Southeast Asia and southwestern China, southeastern Australia, and New Zealand. Most of the abovementioned change rates passed the significant level of 0.1. High spatial positive correlations existed for the variation rates of global HDDs, and both cold and hot spots existed. The vast majority of the global HDDs showed sustainability trends in the future.
(3) The HDDs were significantly positively partially correlated with latitude, altitude, mean albedo, and EVI during winter, and significantly negatively partially correlated with annual mean PM_2.5_ concentration, NTL intensity, and distance to large waterbodies (seas or oceans) (*p* = 0.000). The interannual change rates of HDDs were significantly negatively partially correlated with latitude, altitude, mean albedo during winter, and distance to large waterbodies, and significantly positively partially correlated with the mean EVI during winter, annual PM_2.5_ concentration and NTL intensity (*p* = 0.000).
(4) Both the predicted HDDs and their interannual change rates by GRNN algorithm were significantly highly correlated with their actual values (*p* = 0.000). The RMSEs of HDDs and their variation rates for the testing samples were 564.08 °C * days and 3.59 °C * days * year^−1^, respectively.
(5) Our findings could support the scientific response to climate warming, the construction of living environments, sustainable development, etc. In the future, the global HDDs should be derived from other data, especially the observed data for the related studies. Moreover, more influence factors should be considered, such as the greenhouse gas concentration, atmospheric circulation indices, landscape composition and pattern, remotely sensed indices, the sky view factor, heat release, population density, etc. More accurate and detailed mechanisms should be explored under different contexts at multiple scales during different periods. More effective methods should be adopted, such as experimental observation, numerical simulation, etc.

## Figures and Tables

**Table 1:** Simulation accuracy of heating degree days (HDDs) and their interannual change rates based on the generalized regression neural network algorithm.

Samples	HDDs		Change Rates of HDDs	
	RMSE	R	RMSE	R
Training samples	551.59	0.987 **	3.56	0.881 **
Testing samples	564.08	0.986 **	3.59	0.879 **

** represent being significant at the 0.000 level.

## Data Availability

The data are available upon reasonable request.
